# Effect of *Schistosoma haematobium* infection on the cognitive functions of preschool age children and benefits of treatment from an endemic area in Zimbabwe

**DOI:** 10.1186/s12879-022-07784-7

**Published:** 2022-10-31

**Authors:** Maritha Kasambala, Takafira Mduluza, Arthur Vengesai, Tariro Mduluza-Jokonya, Luxwell Jokonya, Herald Midzi, Rutendo Birri Makota, Arnold Mutemeri, Emmanuel Maziti, Bazondlile Dube-Marimbe, Dixon Chibanda, Francisca Mutapi, Samson Mukaratirwa

**Affiliations:** 1 School of Life Sciences, University of KwaZulu-Natal, KwaZulu-Natal, Durban, South Africa; 2 Department of Biological Sciences and Ecology, University of Zimbabwe, Mt Pleasant, P.O. Box MP 167, Harare, Zimbabwe; 3 Department of Biotechnology and Biochemistry, University of Zimbabwe, Mt Pleasant, P.O. Box MP 167, Harare, Zimbabwe; 4 School of Medicine and Medical Sciences, University of KwaZulu-Natal, KwaZulu-Natal, Durban, South Africa; 5 Department of Biochemistry, Faculty of Medicine, Midlands State University, Senga Road, Gweru, Zimbabwe; 6 Department of Surgery, College of Health Sciences, University of Zimbabwe, Mt Pleasant, P.O. Box MP 167, Harare, Zimbabwe; 7 Department of Psychiatry, College of Health Sciences, University of Zimbabwe, Mt Pleasant, P.O. Box MP 167, Harare, Zimbabwe; 8 Institute for Immunology and Infection Research and Centre for Immunity, Infection and Evolution, School of Biological Sciences, University of Edinburgh, Ashworth Laboratories, King’s Buildings, Charlotte Auerbach Rd, EH9 3JT Edinburgh, UK; 9 One Health Center for Zoonoses and Tropical Veterinary Medicine, Ross University School of Veterinary Medicine, Ross University School of Veterinary Medicine, Basseterre, West Indies Saint Kitts And Nevis

**Keywords:** Cognitive functions, Early child development, Pre-school aged children, Schistosomiasis

## Abstract

**Background:**

Schistosomiasis is known to affect the cognitive functions of children, however, but there is paucity of information on its impact on early childhood development in developing countries where the disease is endemic. This study aimed at determining the effects of schistosomiasis due to *Schistosoma haematobium* on early childhood development in children below 5 years old from Murewa District, Zimbabwe, including the benefits of treatment.

**Methods:**

Preschool age children (PSAC) under the age of 5 years were screened at baseline and at 6 months post-treatment for *S. haematobium* infections diagnosed using the urine filtration method. Cognitive domains were assessed using the Griffith Mental Developmental Scales III on 136 PSAC. Multivariate logistic regression was used to determine the level of association between *S*. *haematobium* infection and performance in the cognitive domains adjusting for confounding factors (i.e. nutrition, hemoglobin levels, gender and age). Median Development Quotient scores of each cognitive domain at baseline and at 6 months post-treatment were compared and quantified.

**Results:**

After adjusting for confounding factors, PSAC infected with *S. haematobium* had greater odds of having lower scores in the Foundation of Learning Domain (OR = 3.9, p = 0.008), Language and Communication Domain (OR = 3.2, p = 0.017), Eye-Hand Coordination Domains (OR = 10.7, p = 0.001), Personal-Social-Emotional Domain (19.3, p = 0.001) and in the Overall General Development Domain (7.2, p = 0.011). Improvement of cognitive performance was observed at 6 months post treatment in the following Domains; Language and Communication Domain (p = 0.003), Eye-Hand Coordination Domain (p = 0.02) and General Development Domain (p = 0.006).

**Conclusion:**

The study showed that *S. haematobium* infection in PSAC is associated with lower cognitive scores in the Foundation of Learning, Language and Communication, Eye-Hand Coordination, Personal-Social-Emotional and in the Overall General Development domains. Our results strengthen the call for inclusion of PSAC in routine deworming programs for the control of urinary schistosomiasis and the need to develop locally validated tools to monitor early child development in endemic areas where resources are limited.

## Introduction

Children growing up in schistosomiasis endemic areas have been disproportionately affected by the disease for decades, as they account for 123 million of the total global burden of over 250 million people infected [1]. Schistosomiasis affects multiple body systems such as the urogenital, gastrointestinal, respiratory and nervous system [2–5]. The impact of infection within the nervous system has been linked to learning difficulties, poor school performance, growth retardation and cognitive deficits [6–8]. The majority of the infected children reside in low-income countries particularly in regions that have poor water supply and sanitation facilities [9–11].

In an effort to reduce schistosomiasis morbidity, national deworming programs have targeted school aged children above the age of five years who are at risk of infection (12). However, to date pre-school age children (PSAC) under the age of five years who are also exposed to infection are excluded from the routine deworming programs and are only treated after confirmed diagnosis [12, 13]. Infection control is hindered in this age group due to existing gaps in research on exposure patterns, unavailability of a paediatric praziquantel formulation [14], impact of infection and the absence of the true global burden of schistosomiasis in the PSAC age group [13]. Inclusion of PSAC as the target population on mass drug administration programs for schistosomiasis treatment can be justified by providing evidence of the negative developmental effects of schistosomiasis infections on cognitive domains [15].

According to the Canadian Council on learning (2010); cognitive development in the first five years of early childhood development is crucial as it has an impact on the future success of the child in school, workplace as an adult, and many other aspects of a healthy fulfilling life. The first five years of childhood is characterized by rapid growth and development of the brain [16]. A meta-analysis conducted in 2018 reported on the negative associations of schistosomiasis and cognitive deficits in school aged going children, particularly in the educational, learning and memory domain [8]. However, to date there is paucity of research that shows the direct and indirect health developmental impact of schistosomiasis on PSAC including the benefits of treatment in this age group. Although the mechanism of how schistosomiasis causes cognitive deficits is unknown, it has been suggested that inflammatory mediators could be the cause of disturbances in the hippocampal neuronal functions and hence affecting working memory consolidation [17–20].

This study thus aimed to bridge the knowledge gap by determining the effect of *S. haematobium* infections on early childhood development and cognitive domains in order to provide substantial evidence base to promote comprehensive national programs for schistosomiasis control in the PSAC age group.

## Methods

### Ethical approval

Permission was granted by the Medical Research Council of Zimbabwe (MRCZ/A/2246 and MRCZ/A/2573), Provincial and District Medical Directors and parents of the participating children. The study goals and methodology were explained in the local language (chi*Shona*) to parents and guardians and written parental consent was acquired prior to recruitment of the PSAC. Recruitment of participants was on a voluntary basis and PSAC were allowed to withdraw at any time during the study. Treatment was administered by a clinician and the children were closely monitored for 4 h post treatment.

### Study area

The study was conducted in Magaya village (Murewa district) which is located in Mashonaland East province of Zimbabwe (17°38′49″S 31°46′39″E). The population in Murewa district is 199,607 and subsistence farming is the main activity in this area and contact with unsafe water is frequent (assessed by questionnaire) due to inadequate and poor safe water facilities. The area is reported to have high *S. haematobium* prevalence (> 50%), low *S. mansoni* and soil transmitted helminths prevalence (< 2%) and low *S. haematobium and S. mansoni* co-infections have been reported [11]. Exposure of the village population to *S. haematobium* infections is through washing, gardening, bathing and collection of water from infected snail infested water sources whilst most of the PSAC are exposed to infections passively (mothers and guardians take their PSAC to infected water contact points while doing their water related chores).

### Study Design and Population

The case control study was conducted on PSAC who were matched according to gender and age at baseline to investigate the effect of *S. haematobium* infections on the cognitive functions of PSAC. The study also employed a 6-month post-treatment follow up on the cases to assess the possible cognitive improvement after treatment. A case was defined as a pre-school aged child who had at least one *S. haematobium* egg in their urine samples following screening with the urine filtration technique and microscopy [21]. Controls were defined as PSAC who did not have *S. haematobium* or *S. mansoni* eggs detected in their urine or fecal samples, respectively. A minimum sample size of 133 (27 cases and 106 controls) was calculated to report 80% power and 95% confidence interval assuming 50% exposure in cases and 22% exposure in controls using Odds Ratio of 3.5.

### Inclusion criteria

One hundred and thirty-six PSAC who met all of the following seven inclusion criteria were enrolled in the study from primary health centers in Magaya, Murewa district; (i) were lifelong residents of Magaya, Murewa district (ii) have never been given anthelminthic treatment for schistosomiasis (iii) were less than 5 years at baseline recruitment (iv) did not have mental and/or physical disabilities (v) did not have *S. mansoni* and/or soil transmitted helminths infection after screening with the Kato-Katz method (vii) written consent obtained from parent to participate in the study. Eleven PSAC (8%) who had developmental challenges (DQ scores below 50) were excluded from the study and referred to a clinical psychologist for further examination and therapy.

### Collection of blood and determination of hemoglobin levels

A clinician collected approximately 5 ml of blood from each PSAC into vacutainer tubes that contained EDTA anticoagulant (BD vacutainer, Fisher Scientific). Blood samples were placed in insulated ice cooler boxes and processed in the laboratory within 4 h. A hematology analyzer (MaxM; Coulter, Fullerton, CA) was used to measure the hemoglobin concentration levels in the blood.

### Anthropometry

The height for age index and weight for age index was used to classify the nutritional status of the PSAC using the WHO child growth standards. Height measurements rounded off to the nearest centimetre (cm) were taken using a stadiometer (Gima®). Each PSAC was weighed in light clothes using an electronic scale (Gima®). Measurement of the mid-upper arm circumference (MAUC) for the diagnosis of malnutrition was done using a MAUC tape (AnthroFlex®). The anthropometric measurements were used to generate Z-scores for the Height-for-Age and Weight-for-Age index using the WHO Anthro software, version 3.0.1(http://www.who.int/childgrowth/en/). PSAC with weight Z-scores < -2 were classified as underweight and if they had Height-for-Age Z-scores < -2 they were classified as stunted.

### Parasitology analysis

Urine and stool samples were collected from each participant over three consecutive days and examined microscopically for the presence of *S. haematobium* eggs using the urine filtration method and the Kato-Katz technique for the detection of *S. mansoni* and soil transmitted helminth eggs [21, 22]. PSAC were classified as infected if at least one *S. haematobium* or *S. mansoni* egg was detected in the urine or stool sample, respectively. PSAC who were found to be infected were treated with praziquantel (PZQ) at a standard dosage of 40 mg/kg body weight by a local clinician.

### Psychological examination

The Griffiths Mental Developmental Scales III for children below 72 months (6 years) was used in this study [23, 24]. The scales measured by this tool were as follows; Eye and Hand Coordination, Personal-Social-Emotional, Language and Communication, Foundations of Learning, Gross Motor Function and Overall General Development. The PSAC were assessed by an experienced clinician who had completed an accredited training course on the Griffiths scales. Developmental Quotient (DQ) scores were calculated using the raw data from each scale and further categorized as dichotomous categorical variables for analysis using the linear regression model. Each scale was analyzed separately in which low performance was categorized for DQ scores less than 88.

### Statistical analyses

Stata Version 16.0 (StataCorp LLC, Texas, USA) was used to analyze the data which was first checked and adjusted for normality. T-test and the Mann Whitney test were used for the analysis of the parametric and non-parametric continuous DQ scores respectively. Multivariate Logistic regression analysis was done to determine the association between *S. haematobium* infection and performance on the cognitive domains. The categorized DQ scores for each cognitive domain were used as dependent variables while infection status was used as the independent variable in the logistic regression model. Each confounding factor (nutritional status, age, gender and hemoglobin levels) were analyzed separately as independent variables in the logistic regression model to determine their independent associations with each cognitive domain. The Wilcoxon matched-pairs signed rank test was used to compare the continuous variable DQ scores of infected children at baseline and at 6 months post treatment timeline. Results with p-values < 0.05 were considered to be statistically significant.

## Results

Characteristics of the study population at baseline before schistosomiasis treatment is shown in Table 1. One hundred and thirty-six PSAC were screened for *S. haematobium* infections and 30 (22%) PSAC were diagnosed with *S. haematobium* infections and none with *S. mansoni* and soil transmitted helminths at baseline. Most of the PSAC (86.7%) had moderate infection intensity with 13.5 mean egg count/10 ml urine of urine. The study had more infected female PSAC (22%) than male PSAC (8%). At 6 months post treatment, only five PSAC had *S. haematobium* re*-*infections.

**Table 1:** Demographic characteristics, infection status with *Schistosoma haematobium* and anthropometry of pre-school aged children from Murewa District, Zimbabwe, who participated in a six-month follow-up study

Variable	Whole cohort (n = 136)	Uninfected (n = 106)	Infected (n = 30)	P- value
Foundations of Learning DQ Mean (SD)	95.6 (18.9)	97.6 (19.1)	88.4 (16.8)	**0.0176**
Language and Communication DQ Median (IQR)	96.3 (20)	98.9 (19.7)	94.2 (21.1)	0.5268
Eye-Hand `Coordination DQ Mean (SD)	101.7 (15.1)	102.7 (14.7)	98.2 (16.1)	0.1470
Personal-Social-Emotional DQ Median (IQR)	107.7 (19)	109.1 (19)	105.5 (23)	0.2324
Gross Motor Function DQ Mean (SD)	107.7 (18.7)	112 (16)	109.5 (25)	0.2667
Overall General Development DQ Mean (SD)	102.2 (12.5)	103.7 (11.9)	97 (13.3)	**0.0085**
Age (months) Median (IQR)	51 (23)	50 (25)	52 (20)	0.0803
BMI Z-score Median (IQR)	-0.63 (1.26)	-0.66 (-1.2)	-0.32 (-1.00)	0.3808
Hemoglobin Median (IQR)	12 (1.1)	12.1 (1.1)	12 (1)	**0.0270**
S. haematobium infection mean eggs/10ml urine (SD)		0	13.5(28.5)	
	%	%	%	P value
Sex:				
Male	73	61.32	26.67	**0.001**
Female	63	38.68	73.3	

SD = standard deviation, IQR = interquartile range, g/dl = grams per deciliter, P-value in bold signifies significance at P < 0.05

### Foundations of Learning Domain

PSAC with *S. haematobium* infection were 3.9 times more likely to have low performance in the Foundations of Learning domain in comparison to uninfected PSAC (p = 0.008) (Table 2). Age was found to be a significant confounder affecting this domain (p < 0.05) indicating that older PSAC were less likely to have lower Foundations of Learning scores than the younger PSAC. The other potential confounders (gender, nutritional status and hemoglobin levels) had no significant associations with PSAC performance in the Foundations of Learning.

### Language and Communication Domain

PSAC with *S. haematobium* infections had 3.2 odds of lower performance in the Language and Communication Domain (p = 0.017) in comparison to uninfected PSAC (Table 2). Age was also found to be significant confounders (p = 0.016) affecting cognitive performance in this domain. Older PSAC were at lesser odds (OR = 0.9) of having lower Language and Communication scores than the younger PSAC. Nutritional status, hemoglobin levels and gender had no significant effects on performance in the Language and Communication Domain.

### Eye and Hand Coordination Domain

PSAC infected with *S. haematobium* had 10.7 times odds of lower performance in the Eye and Hand Domain in comparison to uninfected PSAC (p = 0.001) (Table 2). Age, hemoglobin levels and gender were significant confounders affecting performance in this domain. Older PSAC were at lesser odds (OR = 0.9) of having low scores in the Eye and Hand Coordination Domain. Males PSAC had higher odds (4.9) of having low scores compared to the females. Nutritional status and hemoglobin levels had no significant association with performance in the Eye and Hand Coordination Domain.

### Personal-Social-Emotional Domain

PSAC infected with *S. haematobium* had 19.3 odds of lower performance in comparison to uninfected PSAC (Table 2). Older children had lesser odds (0.9) of performing lower in this domain compared to the younger PSAC. Gender, nutritional status, and hemoglobin levels had no significant associations with performance in this domain.

### Gross Motor Function Domain

The nutritional status of PSAC was a confounder affecting performance in this domain (Table 2). PSAC with lower weight for age scores had greater odds of having lower scores in the Gross Motor Function Domain. Infection status, hemoglobin levels, age and gender had no significant associations with the Gross Motor Function Domain.

### Overall General Development

PSAC with *S. haematobium* infections had their Overall General Development affected, as they had 7.2 odds of lower General Development in comparison to the uninfected PSAC. Older PSAC had lesser odds of having low overall General Development compared to the younger PSAC (Table 2).

**Table 2:** Multivariate analysis results of Griffiths Cognitive Domains of gender, hemoglobin and age on pre-school aged children uninfected and infected with *Schistosoma haematobium*

Variable		FLDQ	LCDQ	PSEDQ	EHCDQ	GDDQ
		AOR (95% CI)	AOR (95% CI)	AOR (95% CI)	AOR (95% CI)	AOR (95% CI)
Gender	Male p value	2.4 (0.6–9.2) 0.207	0.6 (0.1–2.4) 0.432	2.6 (0.4–17.7) 0.318	**19.6 (1.1-356.2)** **0.044**	0.5 (0.6–3.6) 0.469
Nutritional status	p-value	1 (0.5–1.8) 0.976	0.6 (0.3–1.2) 0.155	1.2 (0.4–3.2) 0.718	0.4 (0.2–1.1) 0.071	0.9 (0.4-2.0) 0.776
Hemoglobin	p-value	0.9 (0.4–1.9) 0.753	**0.3 (0.11–0.8)** **0.014**	2.5 (0.8–7.5) 0.114	1.5 (0.4–5.4) 0.500	0.3 (0.1–1.1) 0.068
Age	p-value	**0.9 (0.9-1.0)** **0.003**	**0.9 (0.9-1.0)** **0.023**	0.9 (0.8-1.0) 0.061	**0.9 (0.8-1.0)** **0.03**	0.9 (0.9-1.0) 0.996
S. *haematobium* Infection status	Infected p-value	**4.6 (1.2–17.2)** **0.023**	**3.6 (1.0-13.1)** **0.047**	5.9 (0.9–41) 0.074	**28.2 (2.0-414.6)** **0,015**	5.3 (0.7–36) 0.090

FLDQ = Foundations of Leaning Development Quotient, LCDQ = Language and Communication Development Quotient, PSEDQ = Personal-Social-Emotional Development Quotient, EHCDQ = Eye and Hand Coordination Development Quotient, GDDQ = General Development Quotient AOR = adjusted odds ratio; CI = confidence interval; All AORs quantify the odds of poor performance on cognitive domains adjusted for the effect of confounders (age, hemoglobin level, and nutritional status). Statistical significance is indicated by bold numbers. Reference categories for the gender category are females and for the *S haematobium* infection category are uninfected PSAC

### Griffiths Cognitive Developmental Quotients mean scores at baseline and 6 months post treatment follow up timeline

**Fig. 1:**
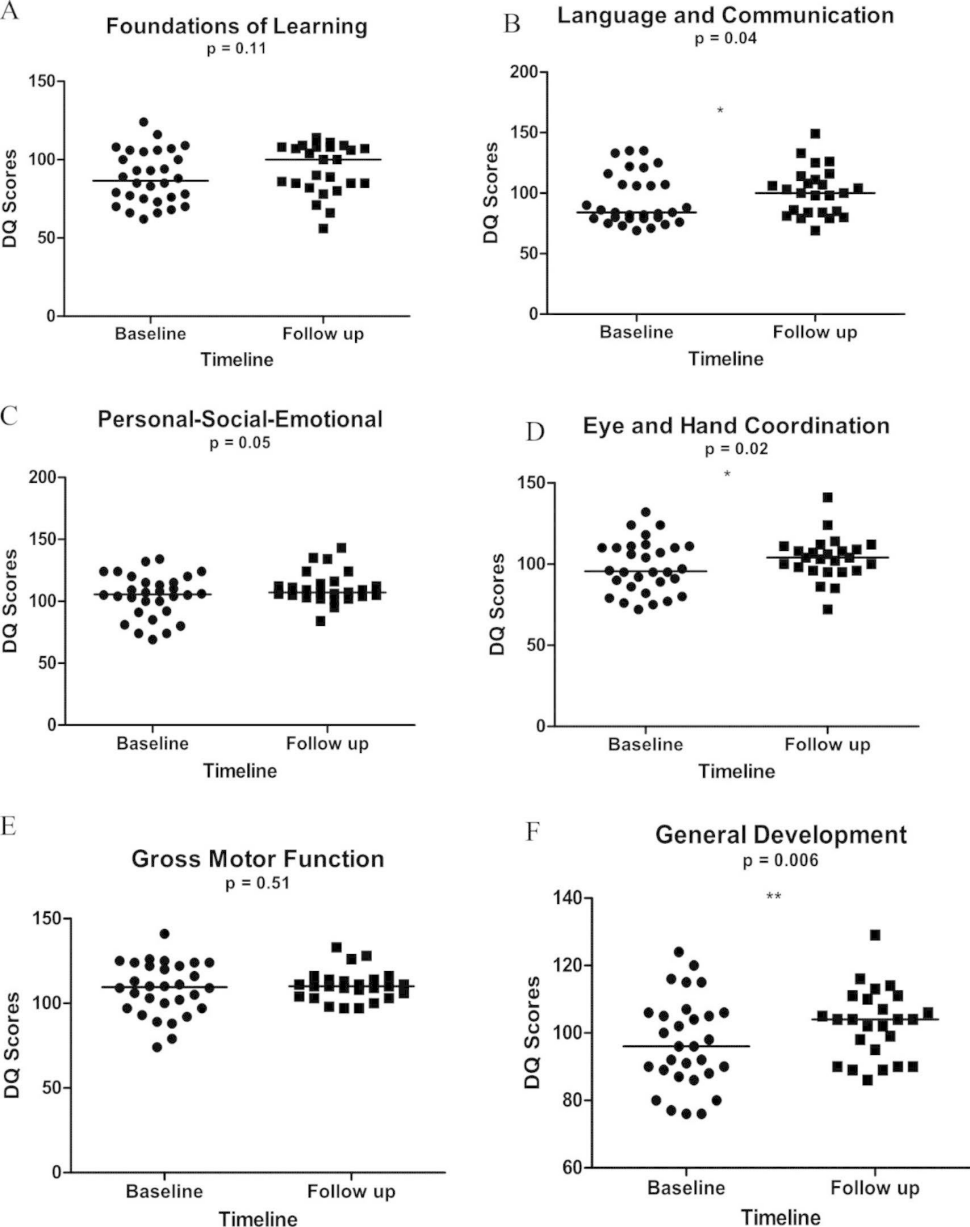
**(A-F )** Comparisons of the Griffiths Cognitive Developmental Quotients median scores at baseline and 6 months post treatment of pre-school aged children (PSAC) infected with *Schistosoma haematobium* (n = 25) using the Wilcoxon matched-pairs signed rank test. A = Foundations of Learning, B = Language and Communication, C = Personal-Social-Emotional, D = Eye and Hand Coordination, E = Gross Motor Function and F = General Development

There was a significant improvement in the PSAC DQ medians (Fig. 1) in the Language and Communication Domain (p = 0.003), Eye-Hand Coordination Domain (p = 0.02) and General Development Domain (p = 0.006) after 6 months post treatment. There was also a non-significant improvement in the cognitive domains; Foundations of Learning, Personal Social-Emotional and Gross Motor Function (p > 0.05).

## Discussion

Various studies have reported on the prevalence of schistosome infections on PSAC, yet the impact on early childhood development has not yet been realized and quantified [25–28]. This study provides evidence base using a Griffith tool of the negative neuro-developmental association of *S. haematobium* infections in PSAC in the Foundations of Learning, Eye and Hand Coordination, Language and Communication, Personal-Social-Emotional and Overall General Development Domain after controlling for gender, age, and nutritional status and hemoglobin levels. The associations between *S. haematobium* infection and the Gross Motor Function were not significant. As this is the first study to our knowledge, reporting on the possible association of *S. haematobium* infections with early childhood development, we compared our results with previous cognition related research that have been done on children above the age of 5 years. Our findings in the Foundations of Learning Domain and Language and Communication Domain are comparable to results from previous studies that reported associations between schistosome infections and poor performance in both the attention, memory and verbal domains in children older than 5 years [29–34].

We hypothesized that poor cognitive performance in the learning and memory domain might be caused by the inflammatory responses against the schistosome worms and or eggs. Both acute and chronic schistosomiasis cause inflammatory responses that are characterized by the production of pro -inflammatory cytokines such as Interferon gamma (IFN-γ), Interleukin-6 (IL-6), Tumor Necrosis Factor (TNF), and C-reactive protein [34–36], [35–37]. These peripheral inflammatory mediators have been reported to be associated with cognitive decline and impairment as they have been reported to be able to affect the brain via different mechanisms and reported to affect working memory [38–41]. The exact mechanism of how inflammation causes cognitive decline and impairment is unknown and there is need for further research on the mechanistic role of inflammation during urinary schistosomiasis on cognitive functions in addition to the characterization of the inflammatory responses and cognitive responses of populations with schistosome infections.

Multiple confounding factors affect cognitive function [42–46], and controlling for all possible confounding factors is challenging in a field-based study which was one of the limitations of this study. We however factored in the most common and important confounders known to influence cognitive function in PSAC such as age, gender and hemoglobin levels (8, 26). In this study, the gender of PSAC had significant associations in the Eye-Hand Coordination Domain where female PSAC performed better than the male PSAC. Our observations are similar to studies which reported that girls in their early childhood stage usually perform better than the boys in different cognitive tests [47, 48]. Contrary to studies reporting the associations of nutritional status and delayed childhood development, socio-emotional, motor and cognitive development [49–56], we did not find any significant association between nutritional status and performance in any of the cognitive domains. In addition, our study did not show significant association of stunting in PSAC with *S. haematobium* infections contrary to studies that were conducted in another district (Shamva) in Zimbabwe [28, 37].

We also report an improvement in the Language and Communication Domain, Eye-Hand Coordination Domain (p = 0.02) and General Development Domain (p = 0.006) after 6 months post treatment emphasizing the importance and positive impact of treatment of schistosomiasis in PSAC. Our results are in agreement to those from a study that was done by Nokes et al. on Chinese primary school children who had *S. japonicum* infections [29].

### Study Limitations

Although the study had an increased ratio of uninfected (controls) to infected (cases) PSAC, we acknowledge the low probability of the presence of undetected schistosome infections in some of the PSAC who were classified as controls. We recommend future studies where larger sample sizes are used to include the use of molecular techniques for the diagnosis of infection parallel to the standard diagnostic techniques used for schistosomiasis.

## Conclusion

Results of this study provides the evidence base of the possible effects of *S. haematobium* infections on the neurodevelopment of PSAC using the comprehensive Griffith Mental Developmental Scales III tool under field conditions. We report significant associations between *S. haematobium* infections and poor performance in the Foundations of Learning, Language and Communication, Personal-Social-Emotional, Eye and Hand Coordination and the Overall General Development Domain in PSAC. Associations between *S. haematobium* infections and the Gross Motor Function Domain were insignificant. Based on the evidence from the study, we recommend that schistosomiasis prevention and control programs consider inclusion of PSAC in the yearly mass drug treatment programs. This will provide cognitive remediation to PSAC residing in schistosomiasis endemic areas. Furthermore, we recommend development and validation of appropriate tools to stimulate foundations of learning for children in resource-limited settings which are endemic for schistosomiasis together with regular treatment of other parasitic infections.

## Data Availability

The datasets used and/or analyzed during the current study are available from the corresponding author on reasonable request.

## Abbreviations

DQ: Developmental Quotient
EHCDQ: Eye and Hand Coordination Development Quotient
FLDQ: Foundations of Leaning Development Quotient
GDDQ: General Development Quotient
GMDQ: Gross Motor Function Development Quotient
IFN –γ: Interferon gamma
IL-6: Interleukin-6
IQR: Interquartile range
MDA: Mass Drug Administration
MRCZ: Medical Research Council of Zimbabwe
PSAC: Preschool aged children
PSEDQ: Personal-Social-Emotional Development Quotient
PZQ: Praziquantel
*S. haematobium*: *Schistosoma haematobium*
TNF: Tumor Necrosis Factor
WHO: World Health Organization
